# Comparative genomic analysis reveals a novel mitochondrial isoform of human rTS protein and unusual phylogenetic distribution of the *rTS *gene

**DOI:** 10.1186/1471-2164-6-125

**Published:** 2005-09-14

**Authors:** Ping Liang, Jayakumar R Nair, Lei Song, John J McGuire, Bruce J Dolnick

**Affiliations:** 1 Department of Cancer Genetics, Roswell Park Cancer Institute, Buffalo, USA; 2 Department of Pharmacology and Therapeutics, Roswell Park Cancer Institute, Buffalo, USA

## Abstract

**Background:**

The rTS gene (*ENOSF1*), first identified in *Homo sapiens* as a gene complementary to the thymidylate synthase (*TYMS*) mRNA, is known to encode two protein isoforms, rTSα and rTSβ. The rTSβ isoform appears to be an enzyme responsible for the synthesis of signaling molecules involved in the down-regulation of thymidylate synthase, but the exact cellular functions of rTS genes are largely unknown.

**Results:**

Through comparative genomic sequence analysis, we predicted the existence of a novel protein isoform, rTS, which has a 27 residue longer N-terminus by virtue of utilizing an alternative start codon located upstream of the start codon in rTSβ. We observed that a similar extended N-terminus could be predicted in all rTS genes for which genomic sequences are available and the extended regions are conserved from bacteria to human. Therefore, we reasoned that the protein with the extended N-terminus might represent an ancestral form of the rTS protein. Sequence analysis strongly predicts a mitochondrial signal sequence in the extended N-terminal of human rTSγ, which is absent in rTSβ. We confirmed the existence of rTS in human mitochondria experimentally by demonstrating the presence of both rTSγ and rTSβ proteins in mitochondria isolated by subcellular fractionation. In addition, our comprehensive analysis of rTS orthologous sequences reveals an unusual phylogenetic distribution of this gene, which suggests the occurrence of one or more horizontal gene transfer events.

**Conclusion:**

The presence of two rTS isoforms in mitochondria suggests that the rTS signaling pathway may be active within mitochondria. Our report also presents an example of identifying novel protein isoforms and for improving gene annotation through comparative genomic analysis.

## Background

The *rTS *(*ENOSF1*) gene, a member of the enolase family, was initially identified in *Homo sapiens *by the discovery of an RNA with extensive complementarity to the mRNA for the DNA biosynthetic enzyme thymidylate synthase[1,2]. The *rTS *gene was later shown to code for two proteins (rTSα and rTSβ) through alternative RNA splicing [2,3]. The mRNA for rTSα is complementary to thymidylate synthase mRNA, while the mRNA for rTSβ is not [2,3]. The rTSβ protein is the major protein product of the *rTS *gene and its expression is associated with the down-regulation of thymidylate synthase protein as cultured cells enter growth arrest [2]. Expression of rTSβ correlates with the production of small molecules that appear to mediate the down-regulation of thymidylate synthase protein by a novel intercellular signaling mechanism [2]. Overproduction of rTSβ occurs in some cells resistant to inhibitors of thymidylate synthase or dihydrofolate reductase, indicating a role for the *rTS *gene in folate and nucleotide metabolism, as well as anticancer drug resistance [2-6].

While the specific function(s) of the *rTS *gene products are currently under investigation, we now report a new rTS protein isoform and its association with mitochondria. The existence of this new isoform, rTSγ, was first predicted using a computational comparative genomic sequence analysis approach and was then verified experimentally. This unexpected observation suggests that rTS may have functions in addition to intercellular signaling.

## Results

### A conserved extended protein N-terminus can be deduced from all available *rTS *genes

**Figure 1:**
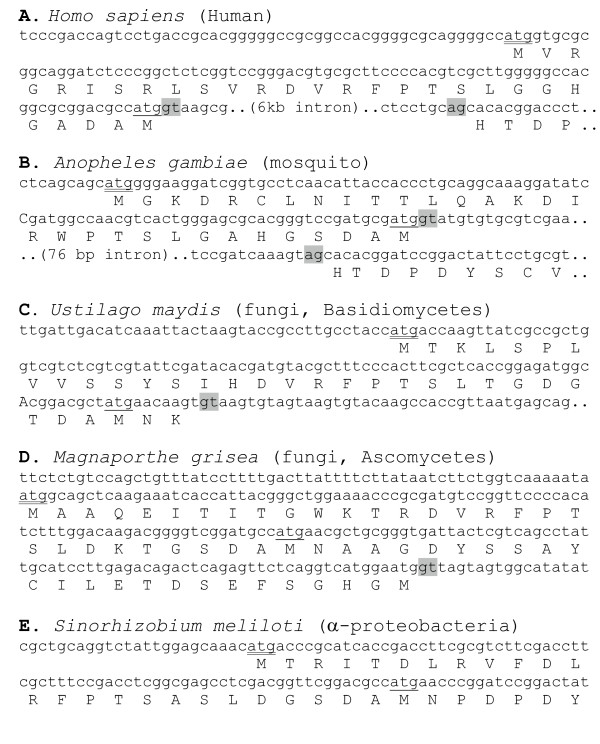
**Prediction of extended N-termini for rTS genes. **The genomic sequences and the predicted protein translations for the N-terminal regions from four different species, *Homo sapiens, Anopheles gambiae, Ustilago maydis*, and *Sinorhizobium meliloti*, are shown in panels A to D, respectively. A double underlined "ATG" indicates the predicted start codon for the extended N-terminus, while the "ATG" with single underline indicates the start codon of the isoform with a shorter N-terminus. Grey highlights indicate the canonical "GT....AG" intron motif.

Comprehensive analysis of all available database sequences revealed that *rTS *genes demonstrate an atypical phylogenetic distribution. *rTS *exists only in a few groups of eubacteria, two fungal lineages (*Ascomycota *and *Basidiomycota*), and most animal species from insects to mammals. Among bacterial *rTS *orthologous genes, several were annotated with a longer N-terminus based on a start codon located further upstream. These proteins include NP_355739.1 (*Agrobacterium tumefaciens str. C58*), NP_540624.1(*Brucella melitensis *16M), NP_639408.1 (*Xanthomonas campestris pv. campestris str*. ATCC 33913), NP_669902.1 (*Yersinia pestis *KIM), NP_828458.1 (*Streptomyces avermitilis *MA-4680), CAD61030.1 (*Arthrobacter ilicis*), and ZP_00227861.1 (*Kineococcus radiotolerans *SRS30216), while many other proteins, including NP_405150.1 (*Yersinia pestis *CO92), NP_437232.1 (*Sinorhizobium meliloti*), NP_533476.1 (*Agrobacterium tumefaciens str*. C58), NP_744975.1 (*Pseudomonas putida *KT2440), ZP_00213853.1 (*Burkholderia cepacia *R18194), ZP_00281771.1 (*Burkholderia fungorum *LB400), AAM39023.1 (*Xanthomonas axonopodis pv. citri str*. 306), and YP_070105 (*Yersinia pseudotuberculosis*) were annotated with an N-terminus equivalent to that of human rTSβ. Therefore, we determined whether an equivalent extended N-terminus could be predicted in the human *rTS *gene. Previously, all available human *rTS *genomic sequences appeared to contain a sequence gap immediately upstream of the start codon of rTSβ, and the published 5'-end of the *rTS *mRNAs was originally determined by RACE (Rapid Amplification of cDNA Ends) analysis of cloned sequences [2]. Thus, a longer N-terminal was not predicted initially, and not expected based upon the existing experimental evidence. However, at the time we started this analysis, one BAC clone, RP11-778P8 [AC021474.1], was found to contain the sequence covering an extended exon region as well as the rest of the gene, although its sequence was in a status of unordered fragments. In addition, a GenBank entry for the human *rTS *gene by Dolnick and Su [AF305057] contains the complete 5'-end upstream sequence. Analysis of this sequence by GenScan [7] predicts a start codon upstream of the start codon for rTSβ, yielding an extended N-terminal that is 27 amino acids longer than rTSβ (Fig. 1A). A sequence comparison between the extended human rTS protein region and the bacterial rTS proteins possessing a longer N-terminus revealed a high level of sequence similarity in the extended region. Therefore, we reasoned that a longer N-terminus may exist in all *rTS *orthologous genes, and this was confirmed by the presence of a possible equivalent extension of the N-terminus in all available *rTS *genomic sequences (Fig. 1A–E). We named this potential new isoform rTSγ to distinguish it from rTSβ and rTSα. During our preparation of this manuscript, NCBI released a new RefGene entry for human rTSβ [NM_202758 and NP_059982.1]. This entry, dated Dec-20-2004, predicted a different N-terminus and was subsequently replaced by another entry [NM_017512 and NP_059982.2] on 02-March-2005, which has a deduced protein product identical to that described in this report. While all available animal *rTS *genes, including those from human, three fish species (fresh and salt water pufferfish and zebrafish), the basal chordate, *Ciona intestinalis*, and the invertebrate *Anopheles gambiae*, share the same intron/exon boundary position at least for the first exon/intron junction (Fig. 1A&B), the fungal *rTS *genes seem to vary in gene structure making them different from the animal genes, as well as from each other (Fig. 1C&D).

**Figure 2:**
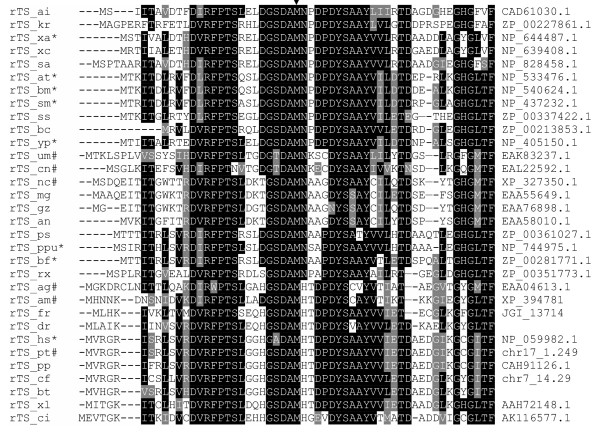
**Sequence conservation within the extended N-terminal regions among all available rTS genes. **The N-terminal section of all available rTS protein sequences were analyzed using Clustal X followed by boxshading using the BOXSHADE server [32]. The arrow indicates the start methionine in the short form of rTS proteins. Black boxed residues are identical, grey boxed residues are similar. The species name of each sequence is indicated by the last two letters in the sequence ID (*ag, Anopheles gambiae; ai, Arthrobacter ilicis; am, Apis mellifera; an, Aspergillus nidulans; at, Agrobacterium tumefaciens; bc, Burkholderia cepacia; bf, Burkholderia fungorum; bm, Brucella melitensis; bt, Bos tauras; cf, Canis familiaris; ci, Ciona intestinalis; cn, Cryptococcus neoformans; dr, Danio rerio; fr, Takifugu rubripes; gz, Gibberella zeae; hs, Homo sapien; kr, Kineococcus radiotolerans; mg, Magnaporthe grisea; nc, Neurospora crassa; pp, Pongo pygmaeus; ppu, Pseudomonas putida KT2440; ps, Polaromonas sp. JS666; pt, Pan troglodytes; rx, Rubrobacter xylanophilus; sa, Streptomyces avermitilis; sm, Sinorhizobium meliloti; ss, Silicibacter sp. TM1040; tr, Takifugu radiatus; um, Ustilago maydis; xa, Xanthomonas axonopodis; xc, Xanthomonas campestris; xl, Xenopus laevis; yp, Yersinia pestis*). The accession numbers of the sequences are provide in the last column of the alignment. Sequences labeled with * are those annotated with a shorter N-terminus, while those labeled with # are the ones containing extra sequences at the N-terminus (see complete sequences in Additional file 1).

In addition to the genomic sequences, EST sequences for *rTS *genes containing sufficient 5'-end sequences were also identified for a few more animal species, including cow, rat, frog, multiple bony fish species, sea squirt, beetles and mites. Multiple sequence alignment analysis revealed that the extended N-termini of *rTS *genes are conserved from bacteria to human (Fig. 2). Therefore, we believe that the extended N-terminal region represents an ancestral form of the *rTS *gene products.

### The extended N-terminus contributes a mitochondrial signal in human rTSγ protein

During our search for potential new motifs and/or functions contributed by the extended N-terminal region of rTS, we found that this extended sequence was predicted to contain a mitochondrial signal. As shown in Table 1, all available programs predict a strong mitochondrial signal for rTSγ, but not for rTSβ, suggesting that the mitochondrial signal is conferred by the extended N-terminal sequence.

**Table 1:** Predicted mitochondrial signal for rTSβ and γ proteins

Program	rTSγ	rTSβ	Reference
Mitoprot	0.9382	0.0117	[22]
TargetP	0.719	0.077	[23]
PSORTII	26.1%	8.7%	[24]
MITOPRED	92.3%	0	[25,26]

### rTSγ protein exists and is associated with mitochondria in human cells

**Figure 3:**
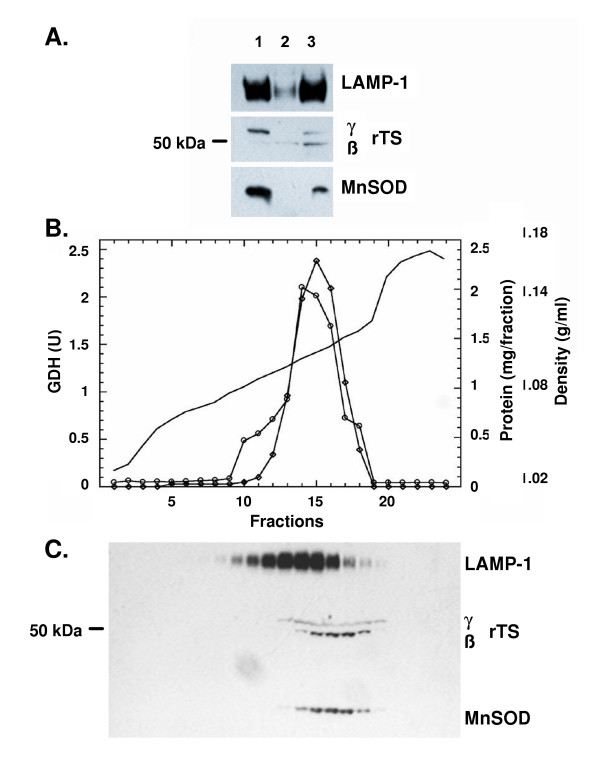
**Localization of rTSβ and rTSγ to mitochondria. A. Presence of rTSβ in both cytosol and organelle fractions. **Five μg of protein from the organelle (lane 1), cytosol (lane 2), or 5 μg each of the organelle plus cytosol (lane 3) were resolved by gel electrophoresis, blotted and probed for the presence of the indicated proteins. **B. Gradient fractionation of mitochondria and lysosomes. **The crude organellar suspension from CCRF-CEM cells was fractionated on a linear iodixanol gradient as described. Individual fractions were collected and assayed for GDH activity and protein content. —, Density (g/ml) of fractions from a mock 5–20% iodixanol gradient; -◊-, GDH activity; -○-, Protein content. **C. Western blot. **Five μl of each fraction was denatured and processed for Western blotting as described.

We addressed the existence of the rTSγ isoform and its possible association with mitochondria experimentally. Initially, a cytosolic fraction and an organellar pellet (including mitochondria and lysosomes) were prepared from CCRF-CEM human cells, and their proteins were resolved by electrophoresis and analyzed for rTS protein expression (Fig. 3A). The results indicate that two rTS proteins with apparent molecular mass of 52.7 ± 1.8 and 47.6 ± 0.7 kDa (approximately corresponding to the difference in the predicted molecular mass of the rTSβ and γ isoforms, respectively) are present. There was a preferential distribution of the higher molecular mass species in the organellar fraction as compared to the cytosolic fraction. The presence in the organellar fraction of both lysosomal (LAMP-1) and mitochondrial (MnSOD) marker proteins, however, did not allow us to conclude that rTS proteins are present in the mitochondria, rather than in the lysosomes. To resolve this, we partially separated lysosomes from mitochondria using a 5–20% iodixanol gradient [8,9](Fig. 3B). The protein profile of the iodixanol gradient shows a peak centered on fractions 14–15 with a pronounced shoulder in fractions 10–12. GDH activity appears in fractions 12–18 with a peak at fraction 15, indicating the presence of mitochondria in these fractions. The shoulder in the protein profile (fractions 10–12) that lacks GDH activity suggests the presence of non-mitochondrial organelles, including lysosomes. This suggestion was confirmed by Western blotting of the gradient fractions for MnSOD and LAMP-1 markers (Fig. 3C). Analysis of the distribution of rTSβ in the gradient indicates that both the rTSβ and γ species co-localize with MnSOD, but not LAMP-1, conclusively demonstrating their presence in mitochondria, but not lysosomes.

## Discussion

### Functional implications for the *rTS *gene based on its mitochondrial location

It has recently been determined that rTSβ is an enzyme responsible for the synthesis of small signaling molecules involved with the down-regulation of thymidylate synthase as cells enter growth arrest *in vitro *[6]. The signaling associated with rTSβ was also shown to act intercellularly [6]. Sequence analysis of the *rTS *gene suggested the presence of a mitochondrial leader sequence that would be expected to increase the length of rTSβ from 416 to 443 amino acids in rTSγ with an expected change in molecular mass from 46,892 to 49,742 Da. Based upon the prediction of a mitochondrial leader sequence, we evaluated whether there is a mitochondrial form of the rTS protein. The results shown in Fig. 3 indicate that this is the case, with two species being present in the mitochondria. These two protein species have apparent molecular masses of 52.7 ± 1.8 and 47.6 ± 0.7 kDa. The smaller species corresponds well to the predicted size expected for rTSβ with the mitochondrial leader sequence cleaved off, while the larger species differs by 3 kDa from the predicted molecular mass for the isoform with the leader sequence. The amounts of rTSγ and rTSβ in the combined cytoplasmic and mitochondrial fractions differ from that expected to result from combining equal amounts of each fraction. The increased abundance of rTSβ, relative to rTSγ in the combined fraction may be the result of *in situ *cleavage of the rTSγ mitochondrial leader sequence by cytosolic proteases, which has been observed to occur in yeast cytosol [10], although transfer of protein may also contribute to the change in signal strength as the MnSOD signal is weaker in the lane with the combined fractions than in the lane loaded with just the organelle protein. Although the migration of proteins in SDS-PAGE gives only approximate estimation of molecular masses, there is a possibility that other post-translational modifications may contribute to this discrepancy. The co-localization of rTSβ and rTSγ with GDH and MnSOD after subcellular fractionation strongly suggests that the extended N-terminal sequence serves as the leader sequence for mitochondrial location of the protein and that it is likely this is partially cleaved to generate the β isoform, once rTSγ is transported into the mitochondria. The presence of the rTSβ protein in mitochondria raises the question of what role this enzyme and the rTS signaling pathway may play there. Mitochondria are a major site for folate metabolism in mammalian cells [11]. Thymidylate synthase is also found within mitochondria [12], despite the absence of a canonical mitochondrial leader sequence, and the relationship of rTS signaling to thymidylate synthase and folate metabolism may ultimately provide the explanation for this phenomenon. Recent evidence indicates that treatment of cells with an rTSβ signaling mimic can affect the cytoskeleton and cause down-regulation of *TYMS *[10,13]. The co-localization of rTS and thymidylate synthase in the mitochondria may indicate that rTSβ, in addition to its role in intercellular signaling, also provides intracellular signals that regulate thymidylate synthase levels in the mitochondria.

Since the extended N-termini of rTS are conserved from bacteria to human, we believe that the rTSγ form of the *rTS *gene represents the ancestral form of this gene, while rTSβ, which seems to be the predominantly expressed form in the cytosol, represents an isoform that appeared later during evolution and came to co-exist with the ancestral isoform, at least in *Homo sapiens*. Based on a recent study which shows that suboptimal AUG codons can support translation via leaky scanning and reinitiation [14], the co-existence of protein products translated using different AUG codons in the same reading frame may not be a rare phenomena. In fact, the starting AUG codons for rTSγ and rTSβ are both qualified as optimal start codons according to recent studies of translation initiation in mammalian and plant genes [14-16], providing an explanation for the co-existence of rTSγ and rTSβ. A recent study suggests that many alternative splicing forms cause differential subcellular localization, especially in targeting either peroxisomes or mitochondria [17], and our data serves as evidence supporting such a notion. An interesting subject for future studies will be to determine when the shorter rTSβ isoform appeared during evolution.

### Phylogenetic distribution and origin of *rTS *gene

**Figure 4:**
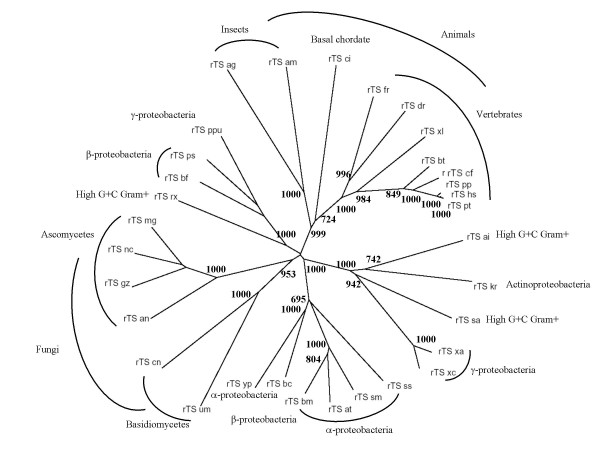
**Unusual phylogenetic distribution of the rTS gene. **The sequences of the same set of proteins displayed in Fig. 2 were used to generate a neighbor-joining tree using ClustalX. The tree was displayed in TreeView program, while the taxonomic labels of the species were added manually afterward. In addition to the extension of the N-terminus as indicated in Fig. 2, adjustments to the currently annotated exon boundaries for a few rTS genes were necessarily made based on the best match between their genomic sequences and the human rTSγ protein sequence (see detailed modifications in supplemental materials). Bootstrap values that are over 700 (1000 trials) are shown at the nodes.

rTS is highly conserved, being found in a variety of species, ranging from bacteria to human. Unlike other enolase genes with a wider phylogenetic distribution, or the thymidylate synthase (*TYMS*) gene, which is ubiquitous in organisms from all three kingdoms and is highly conserved, *rTS *demonstrates an unusual phylogenetic distribution. As shown in Fig. 4, the presence of *rTS *is limited to a few groups of eubacteria including α-, β-, and γ-proteobacteria, actinobacteria, some fungi, and animal species spanning the phylogenetic range from insects to mammals. Among the vertebrate species for which draft genome sequences and/or a large number of EST sequences are available, we were able to identify *rTS *sequences from all species with the exception of chicken and mouse [see a complete list of deduced amino acid sequences in Additional file 1]. Furthermore, we observed in all vertebrate species with sufficient genome sequences including human, chimpanzee, rat, and fugu fish, that the *TYMS-rTS *gene pair is part of a large conserved gene synteny among vertebrates (data not shown). While the absence of *rTS *sequence in chicken may be due to the insufficiency of available genome sequences or ESTs, we were puzzled by the failure to retrieve any *rTS *sequences in mouse, considering the fact that the mouse genome sequence is now fairly complete and its EST data is quite comprehensive. However, we recently obtained preliminary data showing expression of mouse rTS protein and mRNA (data not shown). Another unusual observation is that the *rTS *gene does not seem to be present in *E. coli *while the *rTS *gene is present in another enterobacterium,*Yersinia pestis*. Similarly, although the *rTS *gene is present in *Anopheles gambiae *and several other insect species, but is not found in *Drosophila*. So far, no *rTS *gene has been identified in any plant or yeast species despite the fact that complete genome sequences and comprehensive EST sequences are available for a number of species in these lineages. We also find no rTS sequences from *Caenorhabditis elegans *or other worm genome sequences. The same is true for all archaebacteria and many eubacteria lineages. These observations suggest that the *rTS *gene originated as a bacterial gene which was either horizontally transferred into certain animal and fungal lineages, or alternatively, was lost in all the lineages that do not contain *rTS *genes. An interesting observation is that, while the rTS branches for the animal lineage and the fungal lineage show a topology that agrees well with commonly accepted tree of life for these species, the branches for the bacterial lineages conflict with the commonly accepted tree for the bacterial lineages (Fig. 4). The latter is demonstrated by the fact that species from the same lineage do not always group together, while species from different bacterial lineages do cluster together in many cases (Fig. 4). For example, one β-proteobactial rTS sequence (rTS_bc) is grouped with α-proteobacterial sequences, while the other two β-proteobacterial sequences cluster with a γ-proteobacterial sequence and the three rTS sequences from the High G+C gram positive bacteria are located on three different branches, which all consist of sequences from multiple groups (Fig. 4A). Although not all nodes for the bacterial groups are supported by a high bootstrap value, many nodes are supported with high confidence. This type of unexpected phylotree shown by the rTS sequences suggests the possibility of horizontal gene transfer among the bacteria species, which is common [19-21]. The fact that most of the bacteria and fungi that have a *rTS *gene are human and/or animal pathogens (*Aspergillus nidulans, Brucella suis*, *Brucella melitensis*, *Burkholderia cepacia Burkholderia fungorum, Cryptococcus neoformans, Yersinia pestis, Silicibacter sp)*, plant pathogens (*Agrobacterium tumefaciens*, *Gibberella zeae, Xanthomonas axonopodis*, *Xanthomonas campestris*.*, Ustilago maydis, Magnaporthe grisea*), or in one instance a plant symbiont (*S. meliloti*), adds weight to the hypothesis of horizontal gene transfer events. In the case of plant pathogens, the plants can serve as a mediator between a bacterial donor and an animal acceptor. This may also suggest that the *rTS *gene is required to create and/or maintain a certain type of host-pathogen relationship. Therefore, a better understanding of the biological function of the *rTS *gene may provide new insights for disease control related to these bacterial pathogens.

In addition to the discovery of this novel mitochondrial isoform of rTS, our report also represents a good illustration of the use of comparative genomic analysis for identifying novel protein isoforms of genes and for improving existing gene predictions. Through this study, we have made a comprehensive collection of rTS orthologous sequences and have made a list of suggested modifications to the existing gene annotation (see detailed modifications in the supplemental materials). In addition, by searching all annotated human genes, we identified a few other genes in which evolutionarily conserved alternative upstream AUG codons exist (similar situation as rTSβ). These genes include SELL [NM_000655], SPCS1 [NM_014041.1], NT5C3 [NM_016489], and SDN1 [NM_014390] (data not shown).

## Conclusion

In summary, through comparative genomic analyses we revealed an unusual phylogenetic distribution of the *rTS *gene and identified a novel mitochondria isoform of this gene and verified it experimentally. A mitochondrial location of rTS protein and phylogenetic distribution of this gene provide us with new information that will assist in elucidating its function.

## Methods

### Identification of an extended N-terminal region for all available *rTS *genes

To search for all available *rTS *orthologous sequences, we first queried all protein sequences deposited in the NCBI non-redundant protein database (nr) by BLAST search [21]. Among all identified rTSβ orthologous protein sequences, the ones with an N-terminus equivalent to human rTSβ were identified and their corresponding genomic sequences, including sufficient 5'-end upstream sequences for analysis, were retrieved. Extended N-termini were deduced by extending the start codon to the next available "ATG" upstream of the start codon used by the existing annotation. In addition to the *rTS *orthologous genomic sequences, we also searched the NCBI EST database by performing a TBLASTN search with the extended human rTSβ protein sequence to collect rTS cDNA sequences from additional species. To predict any potential new function or cellular location contributed by the extended N-terminus, we analyzed the human rTSγ and rTSβ protein sequences with Mitoprot [22], TargetP [23], PSORTII [24], and Mitopredict [25,26].

### Subcellular fractionation of CCRF-CEM cells

The human T-lymphoblastic cell line CCRF-CEM was cultured as described [27]. Subcellular fractions of CCRF-CEM cells were isolated essentially as described [28], except that protease (0.5 mM Pefabloc) and phosphatase inhibitors (1 mM sodium metavanadate and 1 mM NaF) were included in all buffers. All steps were performed at 0 – 4°C. Briefly, 1-liter of CCRF-CEM cells (~3.5 × 10^5 ^cells/ml) was centrifuged at 1000 × g for 5 min and the pellet was washed with iced 0.9% NaCl, and then suspended in 5 pellet volumes of ice-cold hypotonic buffer [30] and allowed to swell for 5 min on ice. The suspension was homogenized in an ice-cold 7-ml glass Dounce homogenizer with 15 strokes of the tight pestle to obtain > 95% cell disruption. The homogenate was immediately made up to 250 mM sucrose and centrifuged at 1000 × g for 5 min. The pellet was washed once with 2.5 original pellet volumes of cold isotonic buffer (1 mM Na_2_-EDTA, 250 mM sucrose; pH 6.9). The two supernatants were combined to generate the post-nuclear supernatant (PNS). The PNS was centrifuged at 17,000 × g, 15 min to generate a cytosolic fraction (supernatant) and an organellar pellet containing both mitochondria and lysosomes. The organellar pellet was suspended in 1 ml of HES (20 mM HEPES-NaOH, 1 mM Na_2_-EDTA, 250 mM sucrose; pH 7.4). Subcellular fractions were assayed in duplicate for activity of the cytosolic enzyme lactate dehydrogenase (LDH) [30] and mitochondrial matrix enzyme glutamate dehydrogenase (GDH) [31] to ensure that fractionation was successful. To separate mitochondria and lysosomes, this organellar suspension was made up to 30% (w/v) iodixanol [8,32] in a final volume of 2.2 ml and placed on a 5–20% linear iodixanol gradient (9 ml), centrifuged at 70,000 × g for 1.5 hr, and collected as 0.5-ml fractions, which were then diluted with 0.5 ml ice-cold HES and centrifuged at 30,000 × g for 15 min. The resulting organelle pellets were suspended in 200 μl of isotonic buffer (as above, except that NaF was 50 mM). Protein concentration was determined using the BioRad (Hercules, CA) protein assay kit.

### Western blotting

Proteins were resolved by denaturing gel electrophoresis using 10% polyacrylamide and transferred to PVDF membranes essentially as described [4]. The primary antibodies used were: D3 (mouse monoclonal to rTSβ), LAMP-1 (mouse monoclonal to lysosome-associated membrane protein-1; Santa Cruz Biotechnology), and MnSOD (rabbit polyclonal to manganese superoxide dismutase; Stressgen). Secondary antibodies consisted of horseradish peroxidase conjugated F(ab')_2 _fragments and were obtained from Jackson ImmunoResearch Laboratories. Probed blots were imaged using West Pico Dura chemiluminescent reagent (Pierce) and X-OMAT AR X-ray film (Kodak). Pre-stained protein molecular weight markers (BioRad) were included during electrophoresis to allow determination of apparent molecular weights of detected antigens. All experiments were repeated at least twice with similar results.

## Authors' contributions

PL conceived the project, performed most of the computational analysis, and drafted the complete manuscript; BJD conceived the project and carried out the western blot experiments; JRN and JJM performed the subcellular fractionation and related enzyme assays; LS contributed to the collection and annotations of rTS sequences.

## Supplementary Material

Additional File 1 Deduced rTS protein sequences based on existing DNA sequences and modifications made to existing rTS protein sequences. Click here for file
