# Supplementary material for: Comparative genomic analysis reveals a novel mitochondrial isoform of human rTS protein and unusual phylogenetic distribution of the rTS gene
Source: BMC Genomics. 2005 Sep 14;6:125. doi: 10.1186/1471-2164-6-125 (PMC1261261; doi:10.1186/1471-2164-6-125)
Supplement: Additional File 1 — Deduced rTS protein sequences based on existing DNA sequences and modifications made to existing rTS protein sequences. [file 1471-2164-6-125-S1.doc]

### Additional File 1: Deduced rTS protein sequences based on existing DNA sequences and modifications made to existing rTS protein sequences.

Note: Modifications and the source or basis of protein sequences are provided in the defline. For eukaryotic rTS genes, the protein sequences are divided into number exons where ever the information is available.

>rTS_bm|human rTSgama ortholog|[Brucella melitensis]|gi|17987990|ref|NP_540624.1|N-terminus extended

MTKITDLRVFDLRFPTSQSLDGSDAM

NPDPDYSAAYVILDTDDEALKGHGLTFTIGRGNDICCHAILAMRHLVVGASLDEIRAAPGKFWHHLTGDSQLRWIGPEKGAMHLATGAVVNAIWDLLAKQAGKPVWRLVSELSPEEIADIVDYRYLTDVLTRDEAIEILRKAESGKAERIATLEAEGYACYTTSAGWLGYDDAKLRRLCQEAIDEGFNHVKMKVGHDLEDDIRRLTIAREVIGPDRYLMIDANQVWEVDQAIEWVRRLAFANPFFIEEPTSPDDVAGHRKIRAAIGPVKVATGEMCQNRIMFKQFIAEGAIDVVQIDSCRMGGLNEVLAVLLMAAKYDLPVWPHAGGVGLCEYVQHLSMIDYVAVSGTKEGRVIEYVDHLHEHFLDPCIIRNAAYMPPSLPGFSIEMKPQSIAQYAFKG

>rTS_sm|human rTSgama ortholog|[Sinorhizobium meliloti]|gi|16264440|ref|NP_437232.1|N-terminus extended

MTRITDLRVFDLRFPTSASLDGSDAM

NPDPDYSAAYVILDTDRPGLAGHGLTFTIGRGNDICCMAIEAMRHLVVGQDISNILKHPGRFWRHLTSDSQLRWIGPEKGAIHLATGAIVNAVWDLLAKHAGKPVWRLVADMPAEEIADIVDYRYLTDVLTRDDAVEILRRAEPGKAERIATLEKEGYPCYTTSAGWLGYDDAKLRRLAQEAVDAGFDHIKMKVGRDLDDDIRRLRIAREVIGPDRYLMIDANQVWEVGEAIEWVQKLAFAKPFFIEEPTSPDDVAGHRKIREAIGPVKVATGEMCQNRIMFKQFIAEGAIDIVQIDSCRMGGLNEVLAVLLIAAKYGLPVWPHAGGVGLCEYVQHLSMIDYVAVSGTKDGRVIEYVDHLHEHFLDPCVIRNAAYMPPERPGFSIEMKQQSIEDYRFDG

>rTS_ype|human rTSgama ortholog|[Yersinia pestis]|gi|16121837|ref|NP_405150.1|N-terminus extended

MTIITALRTEDVRFPTSLELDGSDA

MNPDPDYSAAYVILDTDNPALSGHGLTFTIGRGNEICCVAIHALEHLIVGRDLASMTANMGKFWREFTSDSQLRWIGPEKGAIHLATGAVINAVWDLWSKAEGKPLWRLVADMSPEELVRCIDFRYITDCITPEEALTLLQQRAHNKALRLEKLQDEGYPCYTTSAGWLGYPDDKLRRLCQDAVDAGFDYLKLKVGRDLEDDIRRVRITREVLGPDRKLMIDANQIWETNEAIPWVNQLAFANPWFIEEPTNPDDIEGHRRIRQGVAPVKVATGEMCQNRIMFKQFIMREAIDVVQIDACRLGGVNEVLAVMLMAAKYDLPVCPHAGGVGLCEYVQHLAMIDYLCIAGTDKGRVIEYVDHLHEHFVHPCVIKNAAYMPPEQPGFSIEMHQSSIEKYRFRV

>rTS_yps|human rTSgama ortholog|[Yersinia pseudotuberculosis IP 32953]|gi|51595914|ref|YP_070105.1|N-terminus extended

MTIITALRTEDVRFPTSLELDGSDA

MNPDPDYSAAYVILDTDNPALSGHGLTFTIGRGNEICCVAIHALEHLIVGRDLASMTANMGKFWREFTSDSQLRWIGPEKGAIHLATGAVINAVWDLWSKAEGKPLWRLVADMSPEELVRCIDFRYITDCITPEEALTLLQQRAHNKALRLEKLQDEGYPCYTTSAGWLGYPDDKLRRLCQDAVDAGFDYLKLKVGRDLEDDIRRVRITREVLGPDRKLMIDANQIWETNEAIPWVNQLAFANPWFIEEPTNPDDIEGHRRIRQGVAPVKVATGEMCQNRIMFKQFIMREAIDVVQIDACRLGGVNEVLAVMLMAAKYDLPVCPHAGGVGLCEYVQHLAMIDYLCIAGTDKGRVIEYVDHLHEHFVHPCVIKNAAYMPPEQPGFSIEMHQSSIEKYRFRV

>rTS_xa|human rTSgama ortholog|[Xanthomonas axonopodis pv. citri str. 306]|gi|21244905|ref|NP_644487.1|N-terminus extended

MSTIVALDTHDVRFPTSRELDGSDA

MNPDPDYSAAYVVLRTDAADDLAGYGLVFTIGRGNDVQTAAVAALAEHVVGLSVEEVIADLGAFARRLTNDSQLRWLGPEKGVMHMAIGAVINAAWDLAARAAKKPLWRYIAELSPEQLVDTIDFRYLTDALTRDEALAILRAAQPQRAQRIATLIEQGYPAYTTSPGWLGYSDEKLVRLAKEAVADGFRTIKLKVGANVRDDIRRCRLAREAIGPDIAMAVDANQRWDVGPAIDWMRQLAEFDIAWIEEPTSPDDVLGHAAIRQGIAPVPVSTGEHTQNRVVFKQLLQAGAVDLIQIDAARVGGVNENLAILLLAAKFNVRVFPHAGGVGLCELVQHLAMADFVAITGKMEDRAIEFVDHLHQHFLDPVRIRHGRYLAPEAAGFSAEMHAASIAEFSYPGGRFWVEDLAASAKG

>rTS_um|human rTSgama ortholog|[Ustilago maydis 521]|gi|46098004|gb|EAK83237.1|with “SPKDDAEIVSYRGRENEAINAAVKE” and “EMPTGRKQPSAESLVGKNAGPT” removed by splitting exon 2 into two shorter exons

1MTKLSPLVVSSYSIHDVRFPTSLTGDGTDAMNK

2SCDYSAAYLILYT

3DGSLRGFGMTFTIGKGNEICCQAIASIVEELLLGREIEPLFANMGETWQLMVSDPQLRWIGPEKGVVHLATAAVINAIWDLYARSRAKPLWKLICDMSPEELVQCIPFRYITDAITPAEALKILQNAAKGKADREGEMVANGYKAYTTSAGWSGYDDAKVARLTRSALEQGFNHFKLKVGADVADDIRRLSLMRSIVDNPKGCVVMVDANQVWDVQEAIDYMEKLAHLRPWFIEEPTAPDDVLGHAAIRRGIKHLGIGVATGEHAHNRMTFKQLLQADAIDVVQIDSCRLAGVNEILAVMLMAKKFGKIVCPHAGGVGLCEYVVHLSLIDYICISANNERNVLEWVDHLHEHFVYPVSINGAGCYNTPMDAQGGYSIEMLKSSIEDYSFPQGSYWKNGTARTAPPSGH

>rTS_nc|human rTSgama ortholog|[Neurospora crassa]|gi|32413741|ref|XP_327350.1|extra N-terminal sequence removed, the highlighted C-terminal sequence represents another protein fused with rTSgama and is excluded for phylogenetic analysis

1MSDQEITITGWTTRDVRFPTSLDKTGSDAMNAAGDYSAAYCILQTDSKYTGHGM

2TFTIGRGNDIVCSAINHVADRIRGRTLSSLVANWGQTWRHLVNDSQLRWIGPEKGVIHLALGAVVNALWDLWAKVLGKPVWRIVSEMAPEEFVRCIDFRYITDAITPQEAVEMLKETEKTKTERIKDAEANRAVPAYTTSAGWLGYGEDKMRGLLRETMEKGYKHFKLKVGGSVEQDKRRLGIAREVIGYDKGNVLMVDANQVWSVPEAIEYMKELKEFKPWFIEEPTSPDDILGHKAIREALKEYGIGVATGEMCQNRVVFKQLLMSGAIDVCQIDACRMGGVNEVLAVLLIAKKYGVPIVPHSGGVGLPEYTQHLSTIDYVVVSGKKSVLEYVDHLHEHFLYPSVIKDGYYQTPTEPGYSVEMKADSMDRFEYPGGEKSCGYLQNLLLHSLDMYHLDAGIPQRLPPRVLLNKLFRFQDPNIDRLDTSSQPQHSLHTWQVPAQALGAGLNGSVKCQVLRKDLSQLLLGDTMLLSQFHEAALLGMSVALQFQGVPGRQNGVGGDIHDNGSHPVTMPRPSKKAAKPPSDSGEDDHDEHDLSDVEEPPTIDPYEVLGLERDATADQIKTAYRKAALKNHPDKVPAEQKDSATAKFQQIALAYAILSSPTRRQLYDTTGSTSETLASDDGFNWAEYYKSCFADSISPDTIEAFAKSYKNSDEERADVLAAYTDFEGDMDGVYETVMLSDVLEDDERFRTWIDEAIEKKEVDAYPSYTKETKKKRAARVKAAKGEAKEAEELAKELGVYDKLMGSKDGDAKTATKGKAGAKGSKGKKHDGEGALAALILARQQSRGDMFDKLAEKYGAKPKGKGSKRKAEEPPEIDEEEFQRIQAGLGKGSSSSGGTKKAKKRKA

>rTS_ag|human rTSgama ortholog|[Anopheles gambiae str. PEST](African malaria mosquito)|gi|21292468|gb|EAA04613.1|extra N-terminal sequences removed

1MGKDRCLNITTLQAKDIRWPTSLGAHGSDAM0

2HTDPDYSCVYVTIATAEGVTGYGMTFTLGRGTDIVLLAVRAMKRLVEGRTTTSIFERFGQFWRELTSDSQLRW

3IGPEKGVTHLAVAAIINALWDLWGRIRNVPVWQLLAEMEPE

4ELVSTIDFRYIEDVITPEEAIALLRE

5TKPTRSERIQYLLANGYPAYTTQIG

6WLGYSDETIRALCRKYLAAGFKAFKMKVGQDLQNDIKRCKLVREEIGWDNQF

7MIDANQTWNVQTAIEWVISLKDFKPLWIEEPTSPDDVLGQAKIAAALR

8EHSIGVATGEMCCNRVMFKQFMQANALEFCQIDSARIGGVNEILSVYLMAKKLN

9VKVCPHAGGVGLCEMVQHLQMWDFCSVSCTMEGRMVEFVDQQHDQFVFPAAINEQACYVAPRAPGYSTELKQEAILQFEYPHGTEWKRMFDEGIFTRENY

>rTS_am|human rTSgama ortholog|[Apis mellifera](honeybee)|XP_394781|extra N-terminal sequence removed

1MHNNKDNSNIDVKDIRFPTSLLADGSDAM

2HTDPDYSCAYVTIKTKKGIEGYGLTFTLGRGTE

3IVVQACKSMSYLVKGENANNIFTNFGIFWRKLTSESQLRW

4IGPEKGVIHLATAAIINALWDLWARIEKKPVWKLLTDLTPEQLISTIDFR

5YITDVITKEEAIKLLKDNQKGKEEREMILRKNGYPAYTTQVGWLGYSDNKVKELCAKYLALGFTSFKAKVGQNLADDIRRCQLIREVIGYENKLMVDANQIWDINEAIEWMKQLIKFKPTWIEEPTSPDDVLGHAKIANELRPHGIGVATGEMCANRVMFKQLLQARAIDYCQIDSARIGGINEILSVYLMAKKLN

6VPVCPHAGGVGLCEMVQHLQMWDFICLNGSTENRVIEYVDQQHEHFEYPICIQNACYMPPTSPGYSTKFTEDSIKNYSYPNGEKWKNMYKKDSSNLIQII

>rTS_fr|human rTSgama ortholog|Takifugu rubripes (torafugu or pufferfish)|deduced from CAAB01003911.1 (Scaffold_3911)

1MLHKIVKLTVMDVRFPTSSEQHGSDAM

2HTDPDYSAAYVVIETECGLKGFGLTFTLGKGTEI

3VVCAVEALAKLVVGMSWQEIVSDFRGFYRLLTSESQLRWL

4GPEKGVIHLASAAVLNAVWDLWARAEGK

5PLWKLLVDM

6DPKQIVSCIDFRYITDALTEEEAL

7DILLKAREGRQQR

8EDQMLREGYPAYTTSCAWLGYSDELLTQ

9LCTDALQKGWTKFKVKVGADLEDDRRRCRLLREIIGQSNTL

10MIDANQRWDVAEAIRWVSSLAEFNPLWIEEPTCPDDILGHAAISKALAPLGIGVASGEQ

11CQNRVMFKQFLQASALQFVQIDSCRLGSINENLAVLLMAHKFQ

12VPVCPHAGGVGLCELVQHLSLFDYICVSGSLTN

13RMCEYVDHLHEHFASPVVIRNGHYMPPK

14DLGYSCEMLASSVQAHRYPEGDVWKANTSK

>rTS_dr|human rTSgama ortholog|[Danio rerio](zebrafish)|Exon 1-4, 10-11 deduced from CR388017.2, Exon 12-15 supported by EST, CK143066.1, all exons can be deduced from the UCSC genome assembly

1MLAIKIINVSVRDVRFPTSLEQHGSDAM27

2HTDPDYSVAYVVLETDKAELKGYGLTFTVGRGTEI

3VVCAVKALSTLVVGKTLEEITSDFRGFYRLLSSDGQMRWV101

4GPEKGVIHLATAAVLNAVWDLWARVERK129

5PLWKLLVDMDPAK

6LISCIDFRYLTDALTEQEALG 164

7DILVKAKKIRK

8EEQMLKEGYPAYTTSCAWLGYTDQQLTQ204

9LCNEALAQGWTKFKVKVGADLQDDIRRCSLIRKLIGPNNTL244

10MIDANQRWDVNEAITWVTKLAEFQPLWIEEPTCPDDILGHASISK

11ALAPLGIGVASGEQ

12CHNRVMFKQFLQASALQFVQIDSCRVGSVNENLATILMAAKFN345

13VPVCPHAGGVGLCELVQHLILFDYISVSASLSN380

14RMCEFVDHLHEHSKSPTVIRNAKYIPPK

15DPGFSCEMLEESVKKHQYPKGEVWRAIEKQQK439

>rTS_cf|human rTSgama ortholog|[Canis familiaris](dog)|based on chr7_14.29|with exon deleted and exon added based on TBLASTN to the genome sequences

1MVRGRICSLLVRDVRFPTSLGGHGSDAM

2HTDPDYSAAYVVLETDAEDGLKGYGITFTLGKGTEV

3VVCAVNALAHHVLNKDLSDIVGDFRGFYRQLTSDGQLRWI

4GPEKGVVHLATAAILNAVWDLWAKQEGK

5PLWKLLVDMDPRTLLSCIDFRYITDVLTEEEAY

7EILQKGQVGKKER

8EGQMLMHGYPAYTTSCAWLGYSDDTLK

9QLCTEALKAGWT

10RFKVKVGADLQDDVRRCRLIRNMIGPEKTL

11MMDANQRWDVPEAVKWMSKLAEFKPLWIEEPTSPDDILGHATISK

12ALAPLGIGVATGE

13QCHNRVIFKQLLQAKALQFLQIDSCRLGSVNENLSVLLMAKKFEI

14PVCPHAGGVGLCELVQHLIIFDFISISASLQN10

15RMCEYVDHLHEHFRYPVIIKKASYMPPK

16DAGYSTEMKEESVKKHQYPDGEVWKKLLAAQEN

>rTS_pt|human rTSgama ortholog|[Pan troglodytes](chimpanzee)|based on ch17_1.249|extra N-terminal sequence removed and added “FKVKVGADLQDDMRRCQIIRDMIGPEKTL”, exon 6 is missing due to a sequence gap in the genomic sequence

1MVRGRISRLSVRDVRFPTSLGGHGSDAM

2HTDPDYSAAYVVIETDAEDGIKGCGITFTLGKGTEV

3VVCAVNALAHHVLNKDLKDIVGDFRGFYRQLTSDGQLRWIG

4PEKGVVHLATAAVLNAVWDLWAKQEGK

5PVWKLLVDMDPRTLVSCIDFRYITDVLTEEDAL

7EILQKGQVGKKERE

8KQMLAQGYPAYTTSCAWLGYSDDTLK

9QLCAQALKDGWT

10RFKVKVGADLQDDMRRCQIIRDMIGPEKTL

11MMDANQRWDVPEAVEWMSKLAKFKPLWIEEPTSPDDILGHATISK

12ALVPLGIGIATGE

13QCHNRVIFKQLLQAKALQFLQIDSCRLGSVNENLSVLLMAKKFEI

14PVCPHAGGVGLCELVQHLIIFDYISVSASLEN

15RVCEYVDHLHEHFKYPVMIQRASYMPPK

16DPGYSTEMKEESVKKHQYPDGEVWKKLLPAQEN

>rTS_bt|human rTSgama ortholog|[Bos Taurus](cow)|based on mRNA assembled using EST BF074911.1, AJ695692, CF762509.1, CB438135.1, most exons can also be deduced from the UCSC genome assembly

1MVHGRVSRLSVHDVRFPTSLGGHGSDAM

2HTDPDYSAAYVVLETDAEDGLKGYGITFTLGRGTEV

3VVCAVNALAPHVLNKDLGEIVGDFRGFYRQLTSDGQLRWIG

4PEKGVVHLATAAVLNAVWDLWAKQEGK

5PLWKLLVDM

6DPRTLVSCIDFRYITDVLTEEEAC

7EILRQSQVGKKER

8EEQMLAHGYPAYTTSCAWLGYPDATLKQ

9LCSEALKDGWTR

10FKVKVGADLQDDIRRCRLVRNMIGPEKTL

11MMDANQRWDVPEAVEWMTKLAEFKPLWIEEPTSPDDILGHAAISK

12ALAPLGIGVATGEQ

13CHNRVIFKQLLQAKALKFLQIDSCRLGSVNENLSVLLMAKKFE

14IPVCPHAGGVGLCELVQHLIIFDFISVSASLQDR

15MCEYVDHLHEHFKYPVLIREAAYMPPK

16DAGYSTEMKEDSVKRHRYPDGEVWKKLLSAQGN443
